
JOURNAL INFORMATION
==============================
NLM Title Abbreviation: ScientificWorldJournal
Journal ID: ScientificWorldJournal
NLM Journal ID: 101131163
Journal ID: TSWJ

The Scientific World Journal

ISSN: 2356-6140
EISSN: 1537-744X
Publisher: Wiley

ARTICLE INFORMATION
==============================
PMCID: PMC5956474
PMID: 15349546
DOI: 10.1100/tsw.2004.70
Article ID: 765850
Article version: 1
Subjects: Research Article

Laser Surgery of the Prostate: A Review of the Current Options

McNicholas Tom A.
Thompson Alan
Department of Urology, Lister Hopsital, Corey's Miller Lane, Stevenage, Hertfordshire, SG1 4AB, UK
Academic Editor: Anthony Atala

Publication date: 2004
Electronic publication date: 2004 Jun 7
Volume: 4
First page: 201
Last page: 213
Copyright: Copyright © 2004 Tom A. McNicholas and Alan Thompson.
Copyright year: 2004
License: This is an open access article distributed under the Creative Commons Attribution License, which permits unrestricted use, distribution, and reproduction in any medium, provided the original work is properly cited.
License URL: https://creativecommons.org/licenses/by/3.0/

==============================
Articles
*TheScientificWorldJOURNAL* (2004) 4 (S1), 201–213
ISSN 1537-744X; DOI 10.1100/tsw.2004.70

### Laser Surgery of the Prostate: A Review of the Current Options

Tom A. McNicholas, MB, BS, FRCS, FEBU and Alan Thompson, MB, BS, FRCS
*Department of Urology, Lister Hospital, Corey's Mill Lane, Stevenage, Hertfordshire, SG1 4AB, U.K.*

*Previously published in the Digital Urology Journal*

**DOMAIN**: urology

### INTRODUCTION

This article reviews the range of laser techniques available for the treatment of prostatic disease, in particular benign prostatic enlargement (BPE) and prostate cancer. Each modality will be briefly introduced, the mechanism of action and recent experience reviewed and an assessment made of the current and future role of each.

### BENIGN ENLARGEMENT OF THE PROSTATE (BEP)

A quarter of the male population above 40 and 43% of men between 60-69 years, have lower urinary tract symptoms (LUTS) in the presence of a reduced urinary flow rate and an enlarged prostate. 1 Approximately 10-15% of the UK male population will be considered for prostatic surgery at some point in their lives2. Men of 40 years of age in the United States in 1990 appeared to have a 30-40% chance of undergoing prostatectomy if they survived to 80 3 though age-adjusted rates of TURP reached a peak in 1987 and have dropped substantially since as a result of alternative treatments and the onset of “managed care”4.

TURP is regarded as the gold standard in the treatment of bladder outlet obstruction (BOO) secondary to BEP. Immediate relief of symptoms is usual, morbidity acceptable and mortality low5. However, TURP may be associated with long term cardiovascular side effects6 and complications such as impotence7,8and retrograde ejaculation. Consequently there has been growing interest in alternative methods of treatment. This began with microwave therapy and advanced with the development of laser methods. There has now been a return to thermoelectric methods (electro vaporisation) utilising much higher energy levels than used for TURP.

### PROSTATE CANCER

Prostate cancer is the commonest cause of death from malignancy in men in the western world. The main impact of the disease is on older men, 95% of all cases and more than 97% of prostate cancer deaths occurring in men aged 60 and above. Radical prostatectomy or radiotherapy are the de facto primary options for younger men with apparently localised disease in much of the world. Safer or less invasive alternatives or adjuvant therapies will always excite interest. Microwave heating was initially introduced as a treatment for prostate cancer9 then attention turned to BEP10. Cryotherapy, having been explored for BEP in the 1970’s11, has enjoyed a recent revival of interest as an option for prostate cancer12. Focussed ultrasound13 and “Pyrotherapy”14 have potential roles for both benign and malignant prostatic disease. Laser methods initially involved a secondary Neodymium-Yttrium Aluminium Garnet (Nd-YAG) laser coagulation of the prostatic cavity remaining after a “radical” or at least extensive endoscopic resection of the inner prostate15,16, then attention moved to interstitial methods, using the ultrasound techniques developed for radioactive seed implantation17,18 and now photodynamic methods are being actively investigated for both prostate cancer19-23 and for benign prostatic disease24.

### THERMAL THERAPIES IN GENERAL

When tissue is heated there may be minor tissue effects at tissue temperatures between 40 and 44 °C (designated as "Hyperthermia") particularly within malignant tissues25 . But as tissue is heated more (by whatever means) irreversible cellular damage occurs above 45°C. The reciprocal relationship between tissue temperature and duration of heating suggests that if the temperature of target tissue is raised by a few degrees above 45 oC then a cytotoxic effect will be achieved with a much shorter exposure time26. Coagulation necrosis occurs with small and then larger vessel thrombosis leading to later sloughing and/or reabsorbtion of necrotic tissue at temperatures at or above 60°C and vaporisation with acute disruption of tissue due to steam formation occurs at 100°C. Higher temperatures are associated with tissue burning, carbonisation and some immediate tissue removal and tissue disruption due to volatile gas formation.

### NON THERMAL LASER METHODS

### Photodynamic Therapy (PDT)

PDT involves the use of pure light wavelengths to activate previously administered photosensitising agents to cause cell injury by a non-thermal mechanism. Ideally the photo-sensitising drug is taken up or retained to a greater degree by the target tissue than by other tissues. The precise mechanism by which the tumour cells are killed is unclear but probably involves the liberation of oxygen radicals and toxic effects on small blood vessels. 27 In practice there may be a thermal component in some circumstances and there is the possibility of combining PDT and thermal therapies in future. Currently PDT has been more explored as a therapy for prostate cancer rather than for BEP.

Any source of light of the appropriate wavelength and power output may be used for PDT. Endoscopic illumination or interstitial application of the light are both possible. Experiments have been stimulated by the “transparency” of the prostate to certain wavelengths.20 Red light (630 nm) penetrates prostate deeply. PDT has several potential advantages over endoscopic or interstitial laser thermal coagulation.: tissues necrosed by PDT heal with more regeneration and less scarring than after thermal damage so there may be less long term impairment of function .28 It is therefore possible to selectively damage tumour areas whilst preserving adjacent normal tissue exposed to the same sensitizer and light dose, though this requires careful manipulation of parameters . However, systemic exposure to the older, 1st generation photosensitzing agents such as haematoporphyrin derivative can lead to problems, particularly cutaneous photosensitization.

Earlier work using Dunning R 3327 rat prostate cancer cells implanted in Copenhagen rats showed reduced tumour growth in those animals exposed to a photosensitizer and subsequently to red light given interstitially by either single or multiple fibres implanted within the tumour 29though it is possible that a combination of thermal and PDT effects were responsible. A similar effect was shown in vitro where photosensitized Dunning R 3327 cells were killed by exposure to red light at a level that precluded any thermal effects on the cells.30

In the first clinical experience of PDT in the treatment of human prostate cancer Windahl et al.21performed an extended TURP in two patients and then gave a photosensitizer. 48-72 hours later they illuminated the prostatic cavity with 628 nm light through a fibre with a spherical tip. Neither suffered adverse local effects or any of the urgency usually reported following PDT of the bladder. Subsequent biopsies were free of tumour. PSA levels fell from 10 and 6mgm/L to 2.5 and 0.2 mgm/L respectively. One man died of lung cancer and at post mortem was found to have no remaining prostatic cancer. As with endoscopic PDT for bladder cancer and other hollow organ cancers of the body, the most appropriate combination of sensitizer dose and light dose is still being explored.

Increasingly canine prostate experiments have suggested a role for newer photosensitisers such as the chlorins 22 and disulfonated aluminum phthalocyanine (AlS2Pc) and 5-aminolevulinic acid (ALA) - induced protoporphyrin IX. ALA appears to produce small volume effects but AlS2Pc caused more extensive glandular damage while preserving the supporting stromal tissues23.

Possible roles for PDT in prostate cancer may be:

1. For the adjuvant “mopping up” of possibly abnormal tissue just beyond the extent of high temperature or cryotherapy lesions to aid completeness of treatment.
2. To allow treatment of multifocal tumour, probably by means of a multi-fibre interstitial method.
3. For local PDT of an abnormal focal area of prostate, probably by a percutaneous interstitial 1-4 fibre method.
4. By an endoscopic approach, either using a modified fibre or a balloon designed to give adequate and even illumination of the prostatic cavity. This method may be suitable for the man in whom the diagnosis of prostate cancer is made following a TURP.

### LASER THERMAL METHODS

Uptake of laser methods has been rapid but it is important to remember that there are still few properly powered randomised controlled trials (RCTs) from which informed judgements can be made in this evolving subject. Table 1 shows a range of published laser RCTs. Early data on short- term mortality, morbidity, complication rates and outcome is available but we await further details of cost- effectiveness, long- term outcome and patient preference.

### Laser Characteristics and Tissue Effects:

When laser light strikes tissue energy is deposited and tissue is heated. The amount and rate of energy delivery and the degree to which a particular laser wavelength is reflected, absorbed, scattered or transmitted through the target tissue will determine the nature of the thermal process. The power density (PD, the laser power divided by the surface area of the irradiated spot if the laser beam was firing perpendicularly at a flat surface and described as W/cm2) determines the rate of energy and therefore heat deposition onto the target tissue surface (for endoscopic, free beam methods). The Neodymium- Yttrium Aluminium Garnet (Nd:YAG) laser or small, compact Diode lasers with wavelengths causing similar tissue effects (805-980 nanometres) are used for coagulating BPH. Potassium Titanyl Phosphate (KTP) or Holmium lasers (2140nm) produce more avidly absorbed laser wavelengths and have more obviously superficial vaporising and disruptive effects (and so can cut tissue). The same device (or wavelengths) can be used either closer to the tissue to create a high PD with a tendency to a disrupting or “vaporising” effect or held further away to achieve a primarily coagulating effect as desired.

**TABLE 1**

| AUTHORS OF TRIAL | Methods | n = | AUASS pre | AUASS 12 mo | Qmax pre | Qmax 12 mo |
|---|---|---|---|---|---|---|
| Keoghane et al<br>1996<br>42 | Contact tip vs<br>TURP | 72<br>76 | 20.9<br>19.4 | 8.7<br>5.8 | 12.8<br>11.4 | 17.1<br>21.2 |
| Cowles et al<br>1995<br>51 | VLAP vs<br>TURP | 56<br>59 | 18.7<br>20.8 | 9.7<br>7.5 | 8.9<br>9.5 | 14.2<br>16.5 |
| Anson et al<br>1995<br>52 | VLAP vs<br>TURP | 67<br>70 | 18.7<br>18.2 | 7.7<br>5.1 | 9.6<br>10 | 15.4<br>21.8 |
| Narayan et al<br>1995<br>47 | VLAP vs<br>TURP | 32<br>32 | 22.1<br>22.4 | 5.2<br>5.3 | 7<br>6.4 | 16.9<br>19.9 |

Laser light has been applied to the prostate by a series of techniques (Table 2). These methods all show effective prostatic tissue destruction in the largely glandular canine model. However the human gland is far more resistant to heating (by any method). Differing epithelial:stromal ratios could explain differences in response31. Prostatic needle biopsies and transrectal ultrasound (TRUS) images from human laser "failures" had lower epithelial:stromal ratios (1:5) than "successes" (1:3.3) and a more homogeneous Transition zone on TRUS32 raising the possibility of selecting patients for any heat based treatment by TRUS or biopsy.

### *Transurethral endoscopic beam from a simple "bare" fibre*

This is the most basic method of laser surgery to the prostate. The Nd:YAG laser was first widely used in this way during open surgery and then applied to endoscopic surgery. Initially the intention was to achieve coagulation of the target tissue (usually the prostatic lateral lobes33). When more powerful Nd:YAG laser generators or more obviously vaporising wavelengths became available (e.g. Argon, KTP34 and most recently Holmium:YAG35) the techniques of localised vaporisation or cutting of the prostate or bladder neck tissue developed. The simple transurethral endoscopic "bare" fibre is by far the cheapest laser option since no specialised equipment is used but may result in more urethral injury due to the extra manipulation needed to reach all the adenoma. 36

Mattioli37 reported an ingenious mirror adaptation of the albarran lever that deflects the beam from a bare fibre to achieve coagulation of the prostate. He reported efficacy and that this was a reuseable and cost effective device. No further follow up reports are available.

The Holmium:YAG laser fired through a fine fibre close to the tissue surface can cut tissue by vaporisation and can achieve coagulation when held at a distance (or by using a beam deflecting device) thus reducing the power density at the point of beam impact . Development has been rapid: initially the Holmium was used simply as a vaporising addition to Nd:YAG laser coagulation38 but this method was rapidly replaced by purely Holmium vaporisation of the prostate39 or cutting of the bladder neck40 . Now a technique of incising and mobilising the prostatic lobes in a manner similar to resection41 ( intuitively familiar to most urologists) is commanding most attention.

**TABLE 2**

| Method | Effect |
|---|---|
| 1) Transurethral (TU) endoscopic beam from a "bare" fibre.<br>TU endoscopic beam KTP or Holmium laser | Deep coagulation + some vaporisation.<br>Vaporisation + some coagulation. |
| 2) TU endoscopic fibre with a specialised "tip". | Superficial vaporissation or cutting. Some coagulation. |
| 3) Interstitial application of laser energy endoscopically or percutaneously using a "bare" or modified fibre. | Deep localised coagulation with variable immediate tissue disruption (minimal with diffusor tips, more marked with bare fibres). |
| 4) TU endoscopic fibre with various beam deflecting devices.<br>a) TU balloons in combination with deflector.<br>b) Low power density (PD)<br>Wide beam, 40-60W, Non contact.<br>c) High PD Narrow beam, 60-80W, Contact or near contact. e.g. Nd-YAG of Holmium laser. | Deep coagulatoins with minimal immediate tissue disruption.<br>Deep coagulation and some immediate tissue disruption.<br>Variable degree of tissue disruption, some vaporisation with underlying coagulation. |

A bare fibre optimised to carry the holmium wavelenght is protected by a covering ureteric catheter and is passed down a urological endoscope to the prostate. The fibre tip and beam is used to “resect” chunks of the prostatic adenoma from the prostatic capsule. Removing the relatively large pieces of prostatic tissue remains a problem and the protagonists of this development recognise the need to utilise some form of tissue “morcellator” to allow efficient removal of the tissue debris. However, even with these limitations, the early results are very comparable to TURP41 with early catheter removal and minimal postoperative dysuria.

It would be interesting to know the endoscopic and certainly the ultrasonic appearances of these glands after Holmium resection and it is still not clear where all the tissue goes since a relatively small proportion is resected and it is assumed that a similar amount is “vapourised”.

### *Contact laser method: Transurethral endoscopic fibre with a specialised “contact tip”*

The direct application of laser heated probes ( usually synthetic sapphire contact tips, CTs ) is intended to remove enough tissue immediately to allow unobstructed postoperative voiding. The quality of data on CT methods has been strengthened by the Oxford laser prostate trial 42,43 , a double blind RCT of contact tip methods versus TURP which is a good example of a study designed with appropriate statistical powers.

There were no statistically significant differences between the 2 arms in terms of the 7 question American Urological Association symptom score (AUA7SS) response or flow rates at 3 or at 12 months follow up. Other parameters (blood loss, hospital stay and length of catheterisation) favoured the CT laser arm. but significantly more men achieved a large change in AUA7SS (8 pts or more, perceived by the patient as a marked improvement) following TURP.

Catheters were removed 1 night after laser and 2 nights after TURP. Seventeen (28%) men in the CT laser arm failed to void after removal of the catheter compared to 8 (12%) in the TURP arm which suggests that CT methods may allow earlier voiding than coagulative techniques but a significant proportion will still have delay ed voiding . The reoperation rate was 6.6% in the TURP group vs 18.4%

for CT laser. An economic evaluation of the Oxford CT laser prostate trial suggests TURP will remain more cost effective than CT laser methods until the cost of laser consumables is reduced. This well designed study will presumably provide outcome data over the longer term and it may be 5 yrs before we really know the overall failure or retreatment rates which will define the cost effectiveness of this and all other new techniques.

### *The interstitial application of laser energy using a "bare" or modified fibre*

Inserting a small, relatively atraumatic fibre into the prostatic tissue either transurethrally, transrectally or percutaneously under US guidance and heating it up directly (interstitial laser coagulation or ILC) can cause high temperatures and localised coagulative necrosis whilst preserving the prostatic urethral lumen which it is hoped, but not yet proven, may reduce postoperative dysuria following treatment. Since there is no generally agreed system for quantifying post laser symptoms it is difficult to compare one method with another in any meaningful way.

Laser energy can be introduced into prostatic tissue either by:

1. A simple bare fibre which is cheap, but may break.
2. A bare fibre within a cannula through which saline flows to protect the cannula and the fibre. Ultrasound (US) visible changes during prostatic heating allows control of the heating process and follow up of response. In our study of 36 men pre-operative AUA-7 scores of 22 (18 -29) fell to 7.6 (0 - 25 ) and peak flow rates increased from 9.5 (4-14 ) to 14.5 mls/sec (7-32) at 6 months. Patients were taught intermittent clean self catheterisation (ICSC) for an average of 3.7 days (0-7). Clearly, however, not all men benefitted with 4 clearly failing to respond and another 4 responding only moderately. Of those with a good response the average SS change was 17 (8-28), with an average Qmax change of 6.6 mls/second (2-17).44
3. A more complex fibre with a distal "diffusor tip" attached. Over 350 men have been treated by the Munich group since July 1991, by a series of protocols making comparison difficult. Muschter and Hofstetter described the results of several diffusor tip fibres in 239 men treated by the transperineal (n=75) or the transurethral interstitial approach. Twelve month follow up data was available on 127 men. AUA symptom scores (SS) fell from 25 to 6, peak flow rates rose from 8 to 18 mls/sec at 12 months. Irritative symptoms were noted in 12.6%, 4% developed urethral strictures, 7% retrograde ejaculation and no previously potent man developed impotence45.

Preliminary (promotional) data reported for the Indigo diffusor tip fibre and 830 nm diode laser (Indigo, Palo Alto, California, USA) was in line with ILC results outside the German experience. Multicentre RCTs comparing the Indigo diffusor tip fibre and 830 nm diode laser to TURP are underway. One small study has been reported of 25 men treated with this laser and fiber system46. Results included mean SS improvement from 20.6 to 9.4 at 1 month and 6.9 at 3 months. Peak flow rate (Q max) increased from 9.1 to 14.1 and then to 20.3 mls/sec. The system senses temperatures at the fiber tip and adjusts power output with the intention of maintaining a temperature of 100 0C (2120F) throughout the treatment cycle. Temperatures reached and power levels required varied according to the site of puncture with apically placed lesions requiring less energy to achieve and maintain target temperature. Presumably the heat losing mechanisms are less effective at the prostatic apex. Since this report the laser source and system has been "enhanced" so we await new data from studies of the new system before we can really define the effectiveness of the "Indigo" method of ILC.
4. Transurethral endoscopic fibre with various beam deflecting devices. Initially the most popular methods of laser BPH treatment utilised a miniature reflector or prism at the end of the "side-firing" fibre to perform either coagulation ("visual laser ablation" VLAP) or vaporisation (TUVP)

of the prostate. Many operators also coagulated or cut the bladder neck mechanism in order to reduce catheter duration but probably also increasing the incidence of retrograde ejaculation.

### Method of Laser Coagulation (modified visual laser ablation of the prostate or VLAP)

Narayan47 describes a method modified from descriptions of visual laser ablation by Costello48 and Kabalin49. The modified technique consisted of laser energy application at 60 watts for 60 seconds to 11 to 19 spots depending on the size of the prostate. The spots included the 5 and 7 o'clock positions at the bladder neck, the 6 o'clock position for the median lobe, and the 5, 7, 11 and 1 o'clock positions for each 1 cm. length of prostate. For a 3 cm. length of prostate this resulted in 11 spot applications (3 at the bladder neck, and 4 each at the mid and apical prostate areas), while 4 additional spots were used for each additional 1 cm. length. Each spot was wide and covered a 1 cm. area. To ensure coagulation and avoid evaporation, the spot size was enlarged by holding the fiber 2 to 4 mm. away from the tissue. The technique resulted in coagulation of the entire prostatic urethra as visualized by absence of pink mucosa.

Kabalin reported a small prospective RCT where improvements in flow rates and symptom scores in 13 men that equalled a TURP arm and were durable over 18 months followup 50. Larger prospective, multicentre RCTs comparing Nd:YAG VLAP to standard TURP became available and gave more reliable indications of early efficacy even though they were industry sponsored. Table 1 shows SS and Qmax results. A U.S. based study51 enrolled 115 men into 2 trial arms (VLAP by Urolase at 40W, 60 sec, and TURP). The authors concluded VLAP was less effective than TURP but was associated with less “serious” complications (10.7% versus 35.6%) and claimed “non-serious” complications were equal - but only when the higher rate of post VLAP retention and recatheterisation (30.4% vs 8.5%) was excluded.

In a British Urolase study 52 5 centres enrolled 151 men into the 2 trial arms . A similar "spot coagulating"; technique was used but using a higher power (60W power for 60 s). VLAP was associated with significantly less haemorrhage and transfusion and a shorter inpatient stay than TURP (2.7 days vs 4.3) but a longer catheter time (4.8 days vs 2.7). Post operative dysuria was higher at 4 weeks for VLAP (41% versus 15%) and persisted in 15% of men to 3 months compared to only 1% in the TURP group. All remained potent who were so before treatment but there was a surprisingly high incidence of retrograde ejaculation in the VLAP group (33%, compared to 63% for TURP).

At endoscopy at 6 months approximately 40% had residual tissue present , probably due to prudent operators leaving an adequate safety margin at the prostatic apex to avoid stricturing or sphincteric damage. Clearly this untreated tissue may impact on long term outcomes.

These studies revealed encouraging early results and the expected problems of the coagulation method when performed in a variety of centres that probably represented the “real world”. Obviously much longer follow up is needed to show whether these results are durable and the method cost effective. Unfortunately longer term outcomes from these studies have not been reported though presumably the data might still be available. An opportunity to provide the longer term data that we need for the coagulating method may havebeen lost.

The world moves on and even before the results of coagulation methods were clear few urologists were doing the procedure in its simple coagulating form. There was a rapid trend towards using higher energy doses, higher power, higher PD and “vaporising” methods53 in an effort to achieve an immediate tissue defect within the prostatic fossa. In practice the endoscopically obvious “vaporisation” is accompanied by a degree of underlying coagulation as well.

### Method of Laser Vaporisation (TUVP) 47

Before laser evaporation of the prostate standard cystourethroscopy is performed. The limits of laser ablation at the bladder neck and the verumontanum are marked in 4 quadrants at the 8, 4, 11 and 1 o'clock positions. The marks are made 0.5 cm. below the bladder neck and 0.5 cm. proximal to the most prominent portion of the verumontanum.

In all patients initially the laser is applied at the 5 and 7 o'clock positions on the bladder neck at 60 watts until circular fibers of bladder neck are visible. The median lobe enlargement is treated next by laser therapy at a 45-degree angle to the lobe from the right to left sides and vice versa. The ablation is completed by laser application at the 6 o'clock position deep enough to visualize the bladder neck. The laser is used until evaporation of all median lobe tissue is achieved as suggested by the appearance of bladder neck muscle fibers and a smooth, flat bladder neck between the 5 and 7 o'clock positions.

Prostate evaporation is then performed. Four furrows are made from the bladder neck to the verumontanum using the previously delineated landmarks. The furrows at the 8 and 4 o'clock positions are made initially by dragging the fiber on tissue. Tissue evaporation is achieved by holding the laser fiber in contact with the area to be treated and by dragging it at a rate of 1 cm. for every 20 to 30 seconds of laser energy delivery. At the beginning of each furrow dragging is commenced once bubbling is noted signifying evaporation of tissue. Dragging the fiber at a rate of 1 cm./20 to 30 seconds at 60 watts results in a furrow 5 to 7 mm. deep with a 3 to 4 mm. rim of coagulated tissue immediately next to it. After completion of the 4 furrows, a second or third pass is made at the 5 and 7 o'clock positions to determine the depth of the prostate so as to judge the extent of evaporation necessary to obtain satisfactory results. By visualizing the prostatic capsule at some point along the length of the furrow in the lateral lobe (which has the maximal depth), the adequacy of evaporation necessary can be confirmed.

To complete the procedure and establish an open channel, tissues between the furrows (that is between the 11 and 7 o'clock, and the 1 and 5 o'clock positions) are evaporated in a similar manner. The fiber is dragged faster in charred areas (10 seconds per cm.) and slower in coagulated areas (35 to 45 seconds per cm.) because of differences in heat absorption by charred (rapid) versus blanched (slow) tissues. The fiber is also moved at a faster rate (10 seconds per cm.) towards the apex of the prostate where there is less depth to the prostatic lobes.

The procedure is complete when, by visual inspection, a large channel (similar to that after standard transurethral electroresection of the prostate) is created and digital rectal palpation indicates that there is no excessive residual prostate tissue. Any excessive tissue palpated between the cystoscope and rectal finger is evaporated by repeat laser application. The laser is applied to tissue on the floor of the prostate (between the 5 and 7 o'clock positions) only at the median lobe and on either side of the verumontanum. The rest of the tissue is not obstructive and, therefore, laser delivery is avoided, which also probably avoids any inadvertent rectal injury. Hemostasis is achieved by either use of the laser at a lower power setting (20 to 40 watts) and/or moving the fiber away from tissue contact by 2 to 3 mm. A 20F Foley catheter is left indwelling at the end of the procedure in all patients.

Narayan et al. found, in an RCT randomising 64 men between a very high energy vaporising method and a more traditional coagulating technique that though both were effective (Table 1) there was a statistically significantly better flow rate improvement following vaporisation. Coagulated patients were 4 times more likely to develop prolonged postoperative urinary retention (defined as >7 days) and had higher reoperation rates (16% vs 0%)47.

Further data on transurethral laser evaporation of the prostate from the same group includes the results in 168 men with up to 12 months of followup. In fact, of the 168 men reaching 6 months only 129 (76.8%) had reached 12 months of follow up at the time of reporting. Mean age was 68.2 (48-92) and prostatic volume 60.1 (16-132)mls. 37 had a catheter in situ and they usually had major comorbidity. Pre treatment AUASS of 20.6 fell 13.4 (or 65%) to 7.2 (4.1 for the retention group). Pre treatment Qmax of 8.2mls/sec. increased 10mls/sec (or 122%) to 18.2mls/sec. (16.4 for the retention group). 78% had 50% or more improvement in Qmax, 78% had 50% or more improvement in SS. 71% had had 50% or more improvement in both Qmax and SS. Mean energy given was high at 131,281J, or 2,281.7J/cc. of measured prostatic tissue. They used 1.28 fibers per case and men stayed 3.2 days in hospital with a mean catheter duration of 3.4 days. 5 (3%) failed and underwent TURP. 5(3%) voided after a period of delay so were catheterised for 6.8days (5-21d). This group reported irritative symptoms in the 1st month in 32 men (22.6%) for a mean of 22.7days. 1 man developed a stricture during the period of follow up. They concluded that laser evaporation of the prostate was safe and effective but recognised the remaining problems with irritability, suggesting "These symptoms are crippling" and were the main reason patients chose TUEVP or TUNA as an alternative to laser treatment. Multiple fibres were used which added to cost and they reported that laser vaporisation took longer than TURP for a comparable gland ("25-50% longer").

Gilling et al 38, using the Holmium laser in side firing mode, described successful early removal of catheters and very good overall results with this “vaporising” wavelength which can be used either alone or in conjunction with the Nd-YAG laser.

This method was rapidly replaced by purely Holmium vaporisation of the prostate39 with further good results. Despite thes e results this New Zealand group subsequently abandoned coagulative or vaporising methods and further developed Holmium techniques for directly incising and mobilising the prostatic lobes in a manner similar to resection with results exceeding their TURP outcomes41 (see above).

### DISCUSSION

Variability in methods and results is to be expected since we still do not know the answers to many basic questions. We do not even know how much tissue we have to destroy to guarantee a good, long term result though it is probably less than we thought and laser techniques have been shown by pressure flow studies to overcome obstruction despite relatively modest tissue removal compared to a TURP 54-56.

Endoscopic laser prostatectomy by these different methods appears safe. It is usually quicker if one avoids big prostates(>50-60gms). Rapid “barbecueing" or charring of the adenoma surface should be avoided as it leads to high surface temperatures and effects but less deep penetration of light.

Whether size of the prostate is an important consideration is unclear. Jung et al57 reported that VLAP and TURP led to similar symptomatic responses but that VLAP only lowered the “obstructive grade” (measured by pressure flow studies) in those men with prostate size below 50mls whereas TURP was effective for all prostate sizes. Narayan et al. reported that size did not impact on the success of the vaporising method (TUVP) in their experience58.

Similarly, men presenting with acute urinary retention seem to do as well as purely symptomatic men following VLAP59 or TUVP60.

A period of obstruction and “irritability” is common, especially after coagulating laser methods (but can follow any heat therapy). Magnetic resonance(MR) studies immediately following VLAP have shown a mean increase in the volume of the prostate of 34%61,62 which probably contributes largely to the obstruction. It is still not clear to what degree various factors are to blame (such as residual obstruction, infection, the presence of a suprapubic or urethral catheter). ICSC and temporary stents, either absorbable63 or plastic, have been used as alternatives to catheterisation for post laser obstruction. Petas et al. 64 compared the use of a biodegradeable poly-dl-lactic acid (SR-PLA) spiral stent and suprapubic catheter (n=22) to a suprapubic catheter (SPC, n=23) alone following VLAP. Generally voiding was reestablished earlier in those using the stent and SPC so the SPC could be removed earlier. Voiding started on the 1st or 2nd day after VLAP in 18 of 22 with a stent (plus SPC) and in 8 of 23 with the SPC alone. Outcomes (Qmax, SS) were similar at 6 months but the urinary tract infection rate increased with the duration of SP catheterisation. However, stent degradation was prolonged and led to stone formation in 2 men. Clearly, if improved stents prove effective and acceptable to patients then the simpler coagulative method might once again become the leading technique.

Early reports tend to come from enthusiasts and are usually encouraging. Whether this remains true when performed in more routine and widespread urological practice is not yet known. These techniques are more popular in some countries than others and were minimally taken up in general urological practice in the U.K. for instance. Laser methods are reported to be almost equivalent to TURP in terms of symptom response42,51,52. One is led to expect an increase in flow rates of approximately 50% (compared to 100% following a TURP) in an unselected UK population52. Results expressed as means and averages can be misleading and current studies would be more helpful if results were also expressed in terms of generally agreed categories i.e. what proportion obtain an "excellent", "good", "moderate" or "poor" result - if we could agree such categories: such a standardisation is underway but not yet complete 65. McCullough has suggested studies should report the proportion of subjects achieving 50% or greater improvement in Qmax and / or symptoms66.

Kabalin found VLAP a cheaper treatment option67 though Dixon and Lepor, in the first prospective, double blind, laser RCT comparing the safety, efficacy and cost 68, found VLAP no cheaper than TURP in their institution. The Oxford laser RCT found TURP to be more cost effective than contact tip laser methods, mainly due to the cost of consumables69. But if fibers are carefully used more than once70 and if competition among fiber producers intensified then this cost could fall substantially.

### CONCLUSIONS

Despite some excellent reports laser prostatectomy methods in general use do not yet seem as predictably effective in increasing flow rates as TURP though the symptomatic improvement is nearly as good. But the procedures cause little bleeding, are less taxing for the patient, and can be day case procedures - which are considerable advantages. The side firing coagulation method is best known and simplest to use but gives delayed results. New stents might improve outcomes. Data on vaporising methods are now available and are more impressive, leading McCullough to comment that "The vaporization technique has many advantages. Efficacy appears equivalent or nearly equivalent to that of standard transurethral resection of the prostate. An instant channel is created, no bleeding occurs, no absorption of fluid is noted, it can be used on ill patients, including those anticoagulated with heparin, coumadin, nonsteroidal anti-inflammatory drugs and aspirin, no tissue is obtained, no pathology report expense is generated and no postoperative irrigation is required. It deserves wider use"66. Bare fiber Holmium methods of prostatic resection appear to approach the “gold standard” or “yardstick” of TURP but more and longer term data from more centres is needed (and this applies to most of the methods described). The case for contact tip methods has been greatly improved but more long term studies are needed and the cost issue may be crucial. Interstitial methods may well have a role in older, sicker men with larger prostates. ILC remains interesting but unfulfilled due to the lack of good prospective, randomised studies and partly because the technique is still in evolution - which is true of all methods of laser prostatectomy. We need to determine the longevity of the initial response to laser treatment and the incidence of retreatment over many years. Only when we have this data will we be able to define the role of “laser prostatectomy”.

The fundamental challenge to laser techniques is from the “new” TURP, rejuvenated by new vaporising roller or loop technology and improved generator design. The challenge of the laser methods has led to a re-appraisal of what we need to do to relieve symptoms and obstruction in the most cost effective and “patient friendly” manner with the result that significant advances in electrosurgical techniques have ocurred. The explosion of treatment options should not be regarded as a threat but as a development that reinforces the primary role of the urologist as the only health care professional equipped to guide the symptomatic or obstructed man to the best treatment option for him to undergo.

### REFERENCES

1. Garraway, W., Collins, G., and Lee, R. High prevalence of benign prostatic hypertrophy in the community. Lancet, 338: 1991.
2. Neal, D. Evaluation and results of treatments for prostatism. Urol Res, 22: 61, 1994.
3. Barry, M. Epidemiology and natural history of benign prostatic hyperplasia. Urol Clin North Am, 17: 495, 1990.
4. Lu-Yao, G., Barry, M., Chang, C., Wasson, J., and Wennberg, J. Transurethral resection of the prostate among Medicare beneficiaries in the United States: time trends and outcomes. Prostate Patient Outcomes Research Team (PORT). Urology, 44: 692, 1994.

5. Mebust, W., Holtgrewe, H., Cockett, A., Peters, P., , and committee., a. w. Transurethral prostatectomy immediate and postoperative complications. A comparative study of 13 participating institutions evaluating 3,885 patients. J Urol, 141: 243, 1989.
6. Roos, N., Wennberg, J., Malenka, D., Fisher, E., McPherson, K., Andersen, T., Cohen, M., and Ramsey, E. Mortality and reoperation after open and transurethral resection of the prostate for benign prostatic hyperplasia. New Eng J Med, 320: 1120, 1989.
7. Hargreave, T. and Stephenson, T. Potency and prostatectomy. Br J Urol, 49: 683, 1977.
8. Malone, P., Cook, A., Edmonson, R., Gill, M., and Shearer, R. Prostatectomy: patients' perception and long term follow up. Br J Urol, 61: 234, 1988.
9. Yerushalmi, A.: Localised non-invasive deep microwave hyperthermia for the treatment of prostatic tumours: the first 5 years. In: Recent Results in Cancer Research "Application of hyperthermia in the treatment of cancer" . Edited by RD. Issels and W Wilmanns Berlin Heidelberg: Springer Verlag, , pps 141, 1988
10. Devonec, M., Cathaud, M., Carter, S., Berger, N., and Perrin, P. Transurethral microwave application: temperature sensation and thermokinetics of the human prostate. J Urol, 143: 414A., 1990.
11. Green, N. Cryosurgery of the prostate gland in the unfit subject. B J Urol, 42: 10, 1970.
12. Onik, G., Porterfield, B., Rubinsky, B., and Cohn, J. Percutaneous transperineal prostate cryosurgery using transrectal ultrasound guidance: animal model . Urology, 37: 277, 1991.
13. Foster, R. S., Bihrle, R., Fry, F., and Donohoe, J. Transrectal focussed ultrasound ablation of prostate: the first 15 patients. J Urol, 147: 237A (Abstract 93) , 1992.
14. Vallancien, G., Chartier-Kastler, E., Chopin, D., Veillon, B., Brisset, J., and Andre-Bougaran, J. Focused extracorporeal pyrotherapy: experimental results. Eur Urol, 20: 211, 1991.
15. Beisland, H. O. and Sander, S. First clinical experiences on neodymium-YAG laser irradiation of localised prostatic carcinoma. Scand J Urol Nephrol, 20: 113, 1986.
16. McNicholas, T., Carter, S., Wickham, J., and O'Donoghue, E. YAG Laser treatment of early carcinoma of the prostate. Br J Urol, 61: 239 , 1988.
17. Amin, Z., Lees, W., and Bown, S. Technical note: interstitial laser photocoagulation for the treatment of prostatic cancer. Br J Radiol, 66: 1044, 1993.
18. McNicholas, T., Steger, A., and Bown, S. Interstitial laser coagulation of the prostate:An experimental study . Br J Urol, 71: 439, 1993.
19. Gonzalez, S., Arnfield, M. R., Makev, B. E., Tulip, J., Lakey, Q. H., Chapman, J. D., and McPhee, M. S. Treatment of Dunning R3327-At rat Prostate tumours with photodynamic therapy in combination with Misonidiazole. Cancer Res, 46: 2858, 1986.
20. Pantelides, M., Whitehurst, C., Moore, J., King, T., and Blacklock, N. Photodynamic therapy for localised prostate cancer: light penetration in the human prostate gland . J Urol, 143: 398, 1990.
21. Windahl, T., Andersson, S.-O., and Lofgren, L. Photodynamic therapy of localised prostatic cancer (letter). Lancet, 336: 8723, 1990.
22. Chang, S., Buonaccorsi, G., MacRobert, A., and Bown, S. Transurethral and interstitial photodynamic therapy of canine prostate with meso-tetra-(M-hydroxyphenyl) chlorin: applicability and future prospects. J Urol, 155: 354A, 1996.
23. Chang, S., Buonaccorsi, G., MacRobert, A., and Bown, S. Interstitial photodynamic therapy in the canine prostate with disulfonated aluminum phthalocyanine and 5-aminolevulinic acid -induced protoporphyrin IX. Prostate, 32: 89, 1997.
24. Selman, S. Transurethral photodynamic ablation of benign canine prostate. J Urol, 151: 335A, 1994.
25. Song, C. Effect of hyperthermia on vascular functions of normal tissues and experimental tumours. J Natl Cancer Inst, 60: 711, 1978.
26. Moritz, A. and Henriques, F. Studies of thermal injury II. The relative importance of time and surface temperature in the causation of cutaneous burns. Am J Path, 23: 695, 1947.
27. Star, W., Marijnissen, H., van der Berg-Blok, A., Versteeg, J., Franken, K., and Reinhold, H. Destruction of rat mammary tumour and normal tissue microcirculation by haematoporphyrin derivative Photoradiation observed in vivo in sandwich observation chambers. Cancer Research, 46: 2532, 1986.
28. Barr, H., Tralau, C., Boulos, P., MacRobert, A., Tilly, R., and Bown, S. The contrasting mechanisms of collagen damage between photodynamic therapy and thermal injury. Photochem photobiol, 46: 795, 1987.
29. McPhee, M., Thorndyke, C., Tomas, S., Tulip, J., Chapman, D., and Loky, W. Interstitial applications cut laser loss in irradiation of haematoporphyrin derivative photosensitisation. Lasers Surg Med, 4: 93, 1984.
30. Camps, J. J. L., Powers, S. K., Beckman, J. W. C., Brown, J. T., and Weissman, R. M. Photodynamic therapy of prostate cancer: an in vitro study . J Urol, 134: 1222, 1985.
31. Gottfried, H.-W., Vogel, U., and Hautmann, R. Human and canine TULAP - what makes the difference? J Urol, 151: 331A, 1994.
32. Hefty, R., Mattfeld, T., Gottfried, H., Kleinschmidt, K., and Hautmann, R. The critical role of the epithelium-stroma ratio in the laser treatment of BPH. J Urol, 153: 231A, 1995.
33. Camey, M. and Le Duc, A. Preliminary study of the action of the Yag laser on canine prostatic adenoma and experimental urethral stenosis. Eur Urol, 6: 175, 1980.

34. Shanberg, A., Baghdassarian, R., Tansey, L., and Sawyer, D. K.T.P. laser in treatment of urethral strictures. Urology, 32: 517 , 1988.
35. Johnson, D., Cromeens, D., and Price, R. Use of the Holmium:YAG laser in urology. Lasers Surg Med, 12: 353, 1992.
36. Shah, T., Alsudani, M., Akbar, M., Aslam, M., Fowler, L., and McNicholas, T. An inexpensive and effective method of laser ablation of the prostate. J Urol, 153: 414A, 1995.
37. Mattioli, S. Use of bare fiber with siderfiring Albarran bridge in laser therapy of the prostate. J Endourol, 9: no. 2 125, 1995.
38. Gilling, P., Cass, C., Malcolm, A., and Fraundorfer, M. Combination holmium and Nd:YAG laser ablation of the prostate: initial clinical experience. J Endourol, 9: 151, 1995.
39. Gilling, P., Cass, C., Cresswell, M., Malcolm, A., and Fraundorfer, M. The use of the holmium laser in the treatment of benign prostatic hyperplasia. J Endourol, 10: 459, 1996.
40. Cornford, P., Biyani, C., and Powell, C. Transurethral incision of the prostate using the Holmium: YAG laser:a catheterless procedure. J Urol, 159: 1229, 1998.
41. Mackey, M., Chilton, C., Gilling, P., Fraundorfer, M., and Cresswell, M. The results of holmium laser resection of the prostate. BJUrol, 81: 518, 1998.
42. Keoghane, S., Cranston, D., Lawrence, K., Doll, H., Fellows, G., and Smith, J. The Oxford laser prostate trial: A double blind, randomised, controlled trial of contact vaporisation against transurethral resection; preliminary results. Br J Urol, 77: 382, 1996.
43. Keoghane, S., Lawrence, K., Doll, H., and Cranston, D. The Oxford laser prostatectomy trial: one year data from a randomised, controlled trial. B J Urol, 77: Suppl 1 8, 1996.
44. McNicholas, T. and Alsudani, M.: Interstitial laser coagulation therapy for benign prostatic hyperplasia. In: Lasers in surgery: advanced characterisation, therapeutics and systems VI. Edited by R Rox Anderson, Progress in biomedical optics Bellingham, Wa: SPIE- the International Society for Optical Engineering, , 300, 1996
45. Muschter, R. and Hofstetter, A. Interstitial laser therapy outcomes in benign prostatic hyperplasia. J Endourol, 9: 129, 1995.
46. de la Rosette, J., Muschter, R., Lopez, M., and Gillatt, D. Interstitial laser coagulation in the treatment of benign prostatic hyperplasia using a diode-laser system with temperature feedback. BJ Urol, 80: 433, 1997.
47. Narayan, P., Tewari, A., Aboseif, S., and Evans, C. A randomised study comparing visual laser ablation and transurethral evaporation of prostate in the management of benign prostatic hyperplasia. J Urol, 154: 2083, 1995.
48. Costello, A., Bowsher, W., Bolton, D., Braslis, K., and Burt, J. Laser ablation of the prostate in patients with benign prostatic hypertrophy. Br J Urol, 69: 603, 1992.
49. Kabalin, J.: Urolase laser prostatectomy. ed. TA Stamey, 2 ed., Monographs in Urology, vol. 14 (Montverde, Fl: Medical Directions Inc, 1993).
50. Kabalin, J., Gill, H., Bite, G., and Wolfe, V. Comparative study of laser versus electrocautery prostatic resection: 18-month followup with complex urodynamic assessment. J Urol, 153: 94, 1995.
51. Cowles, R., Kabalin, J., Childs, S., Lepor, H., Dixon, C., Stein, B., and Zabbo, A. A prospective randomised comparison of transurethral resection to visual laser ablation of the prostate for the treatment of benign prostatic hyperplasia. Urology, 46: 155, 1995.
52. Anson, K., Nawrocki, J., Buckley, J., Fowler, C., Kirby, R., Lawrence, W., Paterson, P., and Watson, G. A multicentre, randomised, prospective study of endoscopic laser ablation versus transurethral resection of the prostate. Urology, 46: 305, 1995.
53. Shanberg, A., Lee, I., Tansey, L., and Sawyer, D. Extensive neodymium-YAG photoirradiation of the prostate in men with obstructive prostatism. Urology, 43: 467, 1994.
54. James, M., Hariss, D., Ceccherino, A., Manhire, A., and Bates, C. A urodynamic study of laser ablation of the prostate and a comparison of techniques. Br J Urol, 76: 179, 1995.
55. De Wildt, M., Testa, E., Rosier, P., Wijkstra, H., Debruyne, F., and de la Rosette, J. Urodynamic results of laser treatment in patients with benign prostatic hyperplasia. Can outlet obstruction be relieved? J Urol, 154: 174, 1995.
56. Te Slaa, E., De Wildt, M., Rosier, P., Wijkstra, H., Debruyne, F., and de la Rosette, J. Urodynamic assessment in the laser treatment of benign prostatic enlargement. B J Urol, 76: 604, 1995.
57. Jung, P., Mattelaer, P., Wolff, J., Mersdorf, A., and Jakse, G. Visual laser ablation of the prostate: efficacy evaluated by urodynamics and compared to TURP. Eur Urol, 30: 418, 1996.
58. Narayan, P., Tewari, A., Fournier, G., and Toke, A. Impact of prostate size on the outcome of transurethral laser evaporation of the prostate for benign prostatic hyperplasia. Urology, 45: 776, 1995.
59. Farsi, H. Visual laser ablation of the prostate for patients with acute urinary retention. Br J Urol, 78: 90, 1996.
60. Narayan, P., Tewari, A., Schalow, E., Leidich, R., Aboseif, S., and Cascione, C. Transurethral evaporation of the prostate for treatment of benign prostatic hyperplasia: results in 168 patients with up to 12 months of followup. J Urol, 157: 1309, 1997.
61. Flynn, R., de Souza, N., Puni, R., Williams, G., and Kiely, E. Endoscopic laser ablation of the prostate (ELAP): changes in magnetic resonance imaging and clinical outcome at 1 year. Br J Urol, 78: 747, 1996.
62. deSouza, N. M., Flynn, R. J., Coutts, G. A., Gilderdale, D. J., Hall, A. S., Puni, R., Chui, M., Harris, D. N., and Kiely, E. A. Endoscopic laser ablation of the prostate: MR appearances during and after treatment and their relation to clinical outcome. American Journal of Roentgenology, 164: 1429, 1995.

63. Talja, M., Tammela, T., Petas, A., Valimaa, T., Taari, K., Viherkoski, E., and Tormala, P. Biodegradable self-reinforced polyglycolic acid spiral stent in prevention of postoperative urinary retention after visual laser ablation of the prostate - laser prostatectomy. J Urol, 154: 2089, 1995.
64. Petas, A., Talja, M., Tammela, T., Taari, K., Lehtoranta, K., Valimaa, T., and Tormala, P. A randomized study to compare biodegradable self reinforced polyglycolic acid spiral stents to suprapubic and indwelling catheters after visual laser ablation of the prostate. J Urol, 157: no. 1 173, 1997.
65. Aso, Y., Boccon-Gibod, L., Brendler, C., Calais da Silva, F., Cockett, A., Fowler, J., Homma, Y., Moriyama, N., Okajima, E., and Richard, F.: Proposed response criteria. In: The 2nd International Consultation on Benign Prostatic Hyperplasia. Edited by ATK Cockett, et al. Paris, June 27-30 1993: SCI, , 356, 1993
66. McCullough, D. Editorial: Benign Prostatic Hyperplasia--Which Tests Predict Good Outcomes Following Transurethral Resection Of The Prostate And The Role Of Laser Vaporization In Treatment. J Urol, 157: 1313, 1997.
67. Kabalin, J. and Butler, E. Costs of minimally invasive laser-surgery compared with transurethral electro cautery resection of the prostate. West J Med, 162: 426, 1995.
68. Dixon, C., Machi, G., Theune, C., and Lepor, H. A prospective, double blind, randomised study comparing the safety, efficacy and cost of laser ablation of the prostate and transurethral prostatectomy for the treatment of BPH. J Urol, 151: 229A, 1994.
69. Keoghane, S., Lawrence, K., Gray, A., Chappel, D., Hancock, A., and Cranston, D. The Oxford laser prostate trial: economic issues surrounding contact laser prostatectomy. Br J Urol, 77: 386, 1996.
70. Gilbert, H., O'Boyle, P., Mcloughlin, J., and Speakman, M. Multiple use of fibres in the visual laser ablation of the prostate. Br J Urol, 77: 839, 1996.

**This article should be referenced as follows:**

McNicholas, T.A. and Thompson, A. (2004) Laser surgery of the prostate: a review of the current options. *TheScientificWorldJOURNAL* **4 (S1)**, 201–213.

**Handling Editor:**

Anthony Atala, Principle Editor for *Urology* — a domain of *TheScientificWorldJOURNAL*.
